# Exercise capacity in *RYR1*-related myopathies

**DOI:** 10.1186/s13023-025-04013-7

**Published:** 2025-09-24

**Authors:** Lisa M. K. Chin, Joshua J. Todd, Irene C. Chrismer, Jessica W. Witherspoon, Minal Jain, Melissa Waite, Katherine G. Meilleur, Bart Drinkard, Tokunbor A. Lawal

**Affiliations:** 1 Rehabilitation Medicine Department, National Institutes of Health (NIH) Clinical Center, Bethesda, Maryland USA; 2 Neurogenetics Branch, National Institute of Neurological Disorders and Stroke, NIH, Bethesda, Maryland USA; 3 Clinical Trials Unit, National Institute of Neurological Disorders and Stroke, NIH, Bethesda, Maryland USA; 4 Skeletal Myopathies Unit, Translational Biobehavioral and Health Promotion Branch, NIH Clinical Center, Bethesda, Maryland USA; 5 National Institute of Nursing Research, NIH, Bethesda, Maryland USA; 6 NIH Clinical Center, National Institutes of Health, 20814 Bethesda, Maryland USA

**Keywords:** Exercise test, Walk test, Oxygen consumption, Muscular diseases

## Abstract

**Background:**

Pathogenic variations affecting the ryanodine receptor 1 (*RYR1*) gene may result in a variety of neuromuscular disorders, collectively known as *RYR1*-related myopathies. Considered the most common form of congenital myopathy, individuals with *RYR1*-related myopathies may experience skeletal muscle weakness and fatigue, as well as reduced functional capacity. This study examined the exercise capacity in individuals with *RYR1*-related myopathies during a cardiopulmonary exercise test.

**Methods:**

Ambulatory individuals (32 adults, 16 children) with genetically confirmed *RYR1*-related myopathies performed exercise testing on a cycle ergometer and a six-minute walk test at baseline and month six (pre-intervention phase) of a randomized controlled trial (NCT02362425). Outcomes at peak exercise were compared to expected values among the adult and pediatric populations, while longitudinal changes were assessed after six months. Correlations between peak exercise outcomes and the six-minute walk test distance were also examined.

**Results:**

The peak outcomes of oxygen uptake, work rate and heart rate at baseline were lower (all *p* < 0.001) than expected in both adults and children. Peak oxygen uptake expressed as percent predicted was 62 ± 20% and 49 ± 24% in adults and children, respectively. No changes were observed across six months for peak exercise outcomes in either group. A moderately strong positive correlation was observed for peak work rate and six-minute walk test distance among adults (r_s_ = 0.75, *p* < 0.001) and children (r_s_ = 0.64, *p* = 0.008).

**Conclusion:**

Exercise capacity is diminished in adults and children with *RYR1*-related myopathies yet remains stable over six months. The six-minute walk test distance had a direct relationship to peak exercise work rate in adults and children. Exercise capacity testing may be informative for individualizing exercise regimens for persons with *RYR1*-related myopathies. This study was registered with www.clinicaltrials.gov (NCT02362425) on February 12, 2015.

**Supplementary Information:**

The online version contains supplementary material available at 10.1186/s13023-025-04013-7.

## Background

Congenital myopathies (CMyo) include rare neuromuscular disorders often marked by muscular weakness, myalgia, and low exercise tolerance [1, 2]. The most common CMyo are *RYR1*-related myopathies (*RYR1*-RM) [3], with an estimated incidence of 1:90,000 among children in a representative population in the United States [4]. Currently, no approved pharmacological treatments are identified for *RYR1*-RM [5]. The *RYR1* gene is responsible for encoding the ryanodine receptor (RyR1) in the skeletal muscle, and pathogenic variants in the *RYR1* gene result in intracellular calcium dysregulation leading to oxidative stress and, in some cases, decreased RyR1 protein expression [6]. *RYR1*-RM have dominant/de novo and recessive modes of inheritance [7], and most reported variants are classified as variants of uncertain significance [8]. Affected individuals often exhibit generalized muscle weakness and orthopedic issues, however clinical presentation can vary widely [1, 5]. The recessive mode of inheritance in *RYR1*-RM is often associated with greater clinical severity [9], and more severely impacted individuals may require assistance with feeding and respiratory support [1].

Physical exercise is considered crucial for maintaining muscle strength and function, especially in CMyo [1], however the limited number of studies makes exercise recommendations a significant challenge [10]. One interventional study found that a 10-week home aerobic exercise training program improved cardiorespiratory fitness among adults with CMyo, yet had limited effect on functional outcomes [11]. Moreover, magnetic resonance imaging studies elucidated preferential replacement of slow oxidative muscles with non-contractile tissue in the lower extremity of *RYR1*-RM affected individuals [12]. From non-clinical studies, *Ryr1* Y524S knock-in mice exhibited extreme metabolic stress in response to heat exposure, with considerably higher oxygen being consumed (VO_2_) and a switch to anaerobic metabolism (as indicated by higher respiratory exchange ratio, RER, as a ratio of CO_2_ output over O_2_ uptake) when compared to control mice [13]. Pathogenic *RYR1* variants are a common etiology for exertional rhabdomyolysis and heat stroke during intense exercise in affected individuals [14], thus impaired bioenergetics along with exercise intensity and environmental factors are important considerations for exercise in this population.

*RYR1*-RM are largely considered to be a spectrum of slowly progressive neuromuscular disorders [15]. As previously reported, individuals with *RYR1*-RM demonstrated higher oxidative stress and diminished functional capacity compared to reference norms [16], which did not change over 6 months. In *RYR1*-RM, intracellular calcium dysregulation due to leaky RyR1 channels is believed to result in excessive mitochondrial calcium uptake leading to a pathologic skeletal muscle redox state [17, 18]. Thus, it is plausible that the underlying mitochondrial dysfunction coupled with altered lower extremity muscle involvement could result in diminished exercise capacity, as observed in other skeletal muscle disorders [19].

A cardiopulmonary exercise test (CPET) allows examination of an individual’s exercise capacity by conducting a standardized physical test. Exercise is performed at an increasing intensity up to the limit of tolerance or early test termination if clinically indicated [20]. Gas exchange is collected during a CPET for determination of the ventilatory anaerobic threshold (AT) and peak VO_2_, which can serve as prognostic markers for exercise capacity [21]. Recently, the CPET was validated as an appropriate assessment of maximal exercise capacity among adults with neuromuscular diseases [22]. Examination of the dynamic response during the CPET can also further improve our understanding of the dysfunction to muscle metabolism [19] in persons with *RYR1*-RM. Here we characterize exercise capacity and cardiorespiratory function in a cohort of ambulatory adults and children with *RYR1*-RM.

## Methods

### Participants and clinical trial

This investigation was an exploratory analysis of CPET data from the pre-intervention lead-in period of a completed phase II clinical trial (NCT02362425) [16]. The Institutional Review Board (IRB) at the National Institutes of Health (NIH) approved the study protocol, and written informed consent was obtained from all participants prior to enrollment. All study procedures were performed at the NIH Clinical Center in Bethesda, Maryland. This exploratory analysis consisted of ambulatory children (7 to 17 years) and adults (≥ 18 years) with either a confirmed genetic diagnosis of *RYR1*-RM, or a clinical *RYR1*-RM diagnosis with a confirmed *RYR1*-RM genetic diagnosis in a family member. This study consisted of two phases: (1) a six-month prospective natural history study to assess outcome measures in *RYR1*-RM, and (2) another six-month period for a randomized, double-blinded, placebo-controlled trial with oral *N*-acetylcysteine (NAC). Data from the baseline and six-month visit (phase 1) were used for the analyses presented here.

### Physical examination

Details of the physical examination performed and grading of clinical severity for these participants were as described previously [23]. This included a neurological examination on each participant prior to performing the exercise test, to assess their ability to safely perform the CPET.

### Cardiopulmonary exercise test (CPET)

Prior to the CPET, maximal voluntary ventilation (MVV) was determined via spirometry for calculation of breathing reserve. The CPET consisted of exercise on an electronically braked cycle ergometer for adults (or pediatric version, as appropriate), that interfaced with a pulmonary gas exchange system (CardiO_2_ Ultima; MedGraphics Corp, St. Paul, MN). All participants had vitals measured at rest, and continuous monitoring of 12-lead electrocardiogram and heart rate (HR) during exercise. Blood pressure was obtained every two minutes during the CPET. Participants were instructed to sustain a pedal cadence ≥ 60 revolutions per minute (rpm), while resistance was increased by 5 to 10 W/min in children, or 5 to 20 W/min in adults. Ramp rates were chosen based on a participant’s predicted maximal work rate (WR) for sex, age, and weight. The CPET was terminated if the requested pedal cadence could not be sustained, despite verbal encouragement. Peak outcomes were determined as averages of the last 20 s of the CPET. The indices for metabolic, cardiovascular and ventilatory function in adults were also reported from slopes of the VO_2_ vs. WR, HR vs. VO_2_ and ventilation (VE) vs. CO_2_ output (VCO_2_), respectively, using the methods and equations by Neder et al. [24]. The CPET was repeated at the six-month pre-intervention visit using the same ramp rates used at baseline.

As intense exercise may cause exertional rhabdomyolysis or heatstroke in this patient cohort, the CPET was performed in an air-conditioned laboratory, with additional safeguards including a fan blowing on the participant, monitoring of the participant’s temporal temperature and evaluation for the appearance or increase of muscle pain, cramping or stiffness during the test. An affirmative answer for these symptoms or temperature that exceeded 101 degrees Fahrenheit were criteria for immediate termination of the exercise test.

### Ventilatory anaerobic threshold (AT) determination

Identification of the AT from the CPET was made for each participant using accepted gas exchange analysis methods (i.e., the V-slope and ventilatory equivalents) [21]. Independent verification of the AT by the metabolic cart was made by two investigators (BD and LMKC) using multi-panel plots of VO_2_ plotted against VCO_2_, ventilatory equivalents of VO_2_ (VE/VO_2_), ventilatory equivalents of VCO_2_ (VE/VCO_2_), end-tidal O_2_ partial pressure (PET_O2_) and end-tidal CO_2_ partial pressure (PET_CO2_). The AT reflects the point at which energy supplementation from anaerobic sources begins. This was visually observed as the VO_2_ when the following occurred simultaneously [20]: a nonlinear increase in VCO_2_ and VE began, a systematic increase in PET_O2_ without a change in PET_CO2_, and a systematic increase in VE/VO_2_ without an increase in VE/VCO_2_. All AT determinations were further evaluated by two separate investigators (TAL and JJT) and discordant estimations (reviewer variability) were discussed amongst all four investigators until consensus was reached. In the event consensus was not reached, or in cases where the AT could not be ascertained (e.g., test duration was too short, a detectable change was smaller than the data point scatter), such cases were excluded from the AT analysis. The work rate, VO_2_, HR and VE/VCO_2_ at the AT were determined for each remaining participant.

### Six-minute walk test (6MWT)

All participants conducted a 6MWT with instructions to walk with their best effort for six minutes. For safety, participants were accompanied by a physical therapist during the walk, and HR and blood pressure were assessed before, immediately after, and five minutes after the test. Total 6MWT distance provides an indication of overall functional capacity, and primary analyses of the 6MWT have been previously reported for these participants [16, 25].

### Statistical analyses

This study was an exploratory analysis of data acquired during a phase II clinical trial [16]. SPSS (version 29) was used to perform all analyses. Descriptive statistics for categorical variables (i.e., sex and mode of inheritance) are reported as numbers (%), while continuous variables (i.e., age and body mass index) are displayed as mean (±SD). The measured CPET values are reported as mean (±SD) and median (first quartile Q1, third quartile Q3). Normal targets considered for adults [26] during the CPET are provided, where available. Predicted values for CPET variables were determined using published cycle ergometry equations for adults [21, 27] and children [28], and reported as percentage of the predicted value where available. Differences from expected values were calculated (i.e., observed – expected), and normality was assessed using the Shapiro-Wilk test. For parametric and non-parametric distributions, a one-sample t-test of mean difference or a Wilcoxon signed rank test of median difference was used, respectively. Natural history of CPET variables (difference between month-6 and month-0) were also assessed for normality with the Shapiro-Wilk test, and compared with a paired-sample t-test (for parametric distributions) or Wilcoxon signed rank test (for non-parametric distributions). The 95% confidence interval (CI) for mean and median differences, as appropriate, is reported with [lower to upper] bounds. Effect sizes [29] (Hedge’s g, or correlation coefficient r for non-parametric tests) are small (g = 0.20, *r* = 0.10), medium (g = 0.50, *r* = 0.30) or large (g = 0.80, *r* = 0.50) [30]. Due to resistance to outliers, Spearman rank order correlations were used to assess the relationships among peak and AT variables with 6MWT distance, as well as relationships between indices for metabolic, cardiovascular and ventilatory function in adults to peak VO_2_ as % predicted. The strength of the relationship (r_s_) was considered as follows: poor (< 0.3), fair (0.3 to 0.5), moderately strong (0.6 to 0.7) and very strong (≥ 0.8) [31]. Multiple linear regression was used to assess the 6MWT distance (independent variable) and patient characteristics (sex: 0 = male; 1 = female; age in years; BMI in kg/m^2^) as predictors of peak VO_2_ (dependent variable in L/min). Statistical tests are reported as two-tailed analyses.

## Results

Of 53 total enrolled participants, CPET data for 32 adults and 16 children were available at the baseline visit. A detailed clinical assessment of this cohort was reported previously [23]. Briefly, clinical severity of these participants was considered mild to moderate as eligibility was dependent on the ability to perform a 6MWT. Consistent with previous reports [9], clinical manifestations in recessive cases were more severe than dominant/ *de novo* cases, with history of neonatal hypotonia, ptosis, and ophthalmoplegia, along with greater difficulty with ambulation [23]. Baseline characteristics of individuals included in the CPET analyses are shown in Table 1. Both adult and children with *RYR1*-RM completed the CPET without any serious adverse events. Further, none of the tests were terminated for safety concerns, exertional rhabdomyolysis or heatstroke/stress.

**Table 1:** Baseline participant characteristics

	Adult	Child
N	32	16
Age at enrollment in years	19–62 (38.4 ± 12.4)	7–14 (9.3 ± 2.5)
Sex (Male)	14 (43.8)	9 (56.3)
Body Mass Index, BMI in kg/m^2^	10.0–44.0 (25.9 ± 7.5)	11.5–26.5 (16.7 ± 4.7)
Mode of Inheritance, MOI (dominant)	28 (87.5)	10 (62.5)

Data shown as range (mean ± SD) or n (%)

### CPET performance among adults and children: baseline (Month-0)

#### Comparison to predicted values among adults

**Fig. 1:**
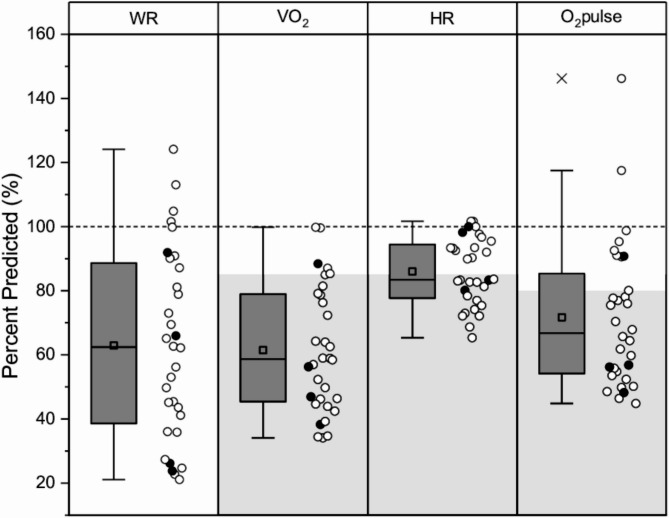
Percent predicted for select peak CPET variables for *RYR1*-RM adults. Standard box plot with ranges (Q1 to Q3), median (line), mean (square symbol), 1.5 times interquartile range (whiskers) and outliers (diagonal cross symbol), and individual participants (circle symbols) shown. Symbols in black denote adults with recessive mode of inheritance. Dashed line denotes expected response (i.e., 100% predicted). Grey areas denote abnormal cut-offs from [20]

The mean cardiorespiratory performance in adults is shown as normative ranges and percent predicted in Table 2 ; Fig. 1, respectively. Compared to normal values [21, 27], CPET outcomes were lower (*p* < 0.001) with large effect sizes (-1.574 ≤ g ≤ -1.091) in adults with *RYR1*-RM. As a group, an acceptable level of peak effort (i.e., RER ≥ 1.10) was attained with peak RER of 1.15 ± 0.12, however peak VO_2_ was only 62 ± 20% of predicted. The average peak work rate was 63 ± 29% of predicted, peak O_2_ pulse (used as a proxy for stroke volume) was 72 ± 23% of predicted, while peak HR reached 86 ± 11% of predicted. At peak exercise, breathing reserve was greater than expected.

**Table 2:** Peak CPET outcome variables in adults (*n* = 32)

	Mean ± SD	Median (Q1, Q3)	Expected value/ Criteria for Normality	Mean Diff [95% CI]	*p*-value	Hedges’s g
Test duration (sec)	672 ± 205	639 (508, 822)	480 to 720	--	--	--
RER	1.15 ± 0.12	1.15 (1.08, 1.23)	≥ 1.10^b^	--	--	--
WR (watts)	90 ± 49	83 (55, 117)	155 ± 56^c^	-65 [-86 to -44]	< 0.001	-1.091
VO_2_ (L/min)	1.39 ± 0.61	1.27 (0.98, 1.47)	2.32 ± 0.71^d^	-0.92 [-1.13 to -0.72]	< 0.001	-1.574
VO_2_ (ml/kg/min)	19.9 ± 6.1	19.0 (16.8, 23.1)	34.6 ± 12.0	-14.7 [-18.4 to -11.0]	< 0.001	-1.395
HR (beats/min)	156 ± 23	156 (142, 176)	182 ± 12^c^	-26 [-33 to -19]	< 0.001	-1.389
HR reserve (beats/min)	26 ± 18	28 (10, 38)	< 15^**b**^	--	--	--
O_2_ pulse (ml/beat)	8.9 ± 3.1	8.5 (6.7, 10.2)	12.7 ± 3.6^c^	-3.8 [-4.9 to -2.7]	< 0.001	-1.209
Ventilation (L/min)	48.7 ± 23.8	40.8 (32.3, 61.3)	91.9 ± 24.0^c^	-43.2 [-53.3 to -33.0]	< 0.001	-1.495
^a^Breathing reserve (%)	50.9 ± 20.0	58.3 (39.8, 65.4)	≥ 15 to 20^b^	--	--	--
Respiratory rate (breaths/min)	36.5 ± 12.8	34.3 (27.3, 44.8)	≤ 50^b^	--	--	--

^a^Breathing reserve calculated as: (MVV-peak VE/MVV)×100. --, not known or not performed. Source for criteria or calculation of predicted values as: ^b^ [26],^c^ [21],^d^ [27]

Examination of slope profiles compared to expected values in adults revealed medium to small effect sizes for ventilatory (*p* = 0.027, *r* = 0.390), metabolic (*p* = 0.054, *r* = 0.340), and cardiovascular indices (*p* = 0.079, g = 0.314) (Supplementary Table 1). However, a moderately strong negative correlation was observed for percent predicted peak VO_2_ and the slope for ∆HR/∆VO_2_ (*p* < 0.001, r_s_ = -0.563) (Supplementary Fig. 1), where adults with more severe exercise capacity impairment demonstrated higher cardiovascular need than expected. Only poor relationships were observed between percent predicted peak VO_2_ and slope for WR/VO_2_ (*p* = 0.670, r_s_ = -0.078), and slope for VE/VCO_2_ (*p* = 0.116, r_s_ = -0.283). Responses to the CPET by two representative females of similar age are shown in Supplementary Fig. 2.

Outcomes at the AT were also assessed and reported in 30 adults (Table 3 ). The VO_2_ at the AT was 36 ± 9% of predicted peak VO_2_, and 22 of the 30 adults (73%) did not reach the expected normal VO_2_ at the AT of ≥ 40% predicted peak VO_2_. Ventilatory efficiency (i.e., Ve/VCO_2_) assessed at the AT was within normal limits for the group.

**Table 3:** Anaerobic threshold (AT) outcome variables in adults (*n* = 30)

	Mean ± SD	Median (Q1, Q3)	Criteria for Normality/ Reference range
WR (watts)	47.8 ± 28.8	36.5 (26.0, 63.3)	--
VO_2_ (L/min)	0.81 ± 0.28	0.72 (0.63, 0.84)	--
VO_2_ (% predicted peak)	36.0 ± 8.7	35.3 (30.3, 40.6)	≥ 40–80
HR (beats/min)	117 ± 20	114 (102, 131)	--
VE/VCO_2_	27.3 ± 3.1	28.4 (25.0, 30.0)	25–30

--, not known. Source for criteria by [26]

#### Comparison to predicted values among children

The mean cardiorespiratory performance in children is shown in Table 4. Among those that performed the CPET, twelve (75%) did not reach an RER ≥ 1.10. Low values with large effect sizes in all CPET outcomes (*p* < 0.001, -2.419 ≤ g ≤ -0.879) were observed in children with *RYR1*-RM compared to normal values from Burstein et al. [28]. The AT was not reported in children due to the relatively short test duration and few reaching an RER ≥ 1.10.

**Table 4:** Peak CPET outcome variables in children (*n* = 16)

	Mean ± SD	Median (Q1, Q3)	Expected value	Mean/Median^a^ Diff [95% CI]	*p*-value	Effect size (g or *r*^a^)
Test duration (sec)	392 ± 213	353 (229, 508)	480 to 720	--	--	--
RER	0.99 ± 0.10	0.95 (0.93, 1.10)	≥ 1.10	--	--	--
WR (watts)	33 ± 18	30 (17, 42)	102 ± 50	-61 [-88 to -44]^a^	< 0.001	-0.879^a^
WR (% pred)	34.1 ± 17.5	27.4 (22.7, 45.4)	--	--	--	--
VO_2_ (l/min)	0.60 ± 0.32	0.52 (0.35, 0.73)	1.35 ± 0.67	-0.74 [-1.06 to -0.43]	< 0.001	-1.203
VO_2_ (%pred)	48.6 ± 23.5	45.4 (28.7, 63.8)	**--**	**--**	**--**	**--**
HR (beats/min)^b^	144 ± 24	140 (122, 159)	194 ± 2	-50 [-63 to -38]	< 0.001	-2.071
HR (% pred)^c^	74 ± 12	73 (63, 82)	--	--	--	--
O_2_ pulse (ml/beat)^b^	4.5 ± 1.5	4.2 (3.7, 5.4)	7.0 ± 3.5	-1.9 [-3.9 to -1.2]^a^	< 0.001	-0.879^a^
O_2_ pulse (% pred)^b^	67.5 ± 15.8	71.0 (51.9, 77.1)	**--**	**--**	**--**	**--**
VE (L/min)	20.4 ± 9.8	18.8 (14.8, 22.9)	62.2 ± 55.9	-41.8 [-50.5 to -33.0]	< 0.001	-2.419
VE (% pred)	33.0 ± 14.1	28.9 (26.0, 35.3)	--	--	--	--
RR (breaths/min)	41.7 ± 11.0	40.3 (32.2, 48.4)	62.8 ± 3.6	-21.2 [-26.9 to -15.4]	< 0.001	-1.864
RR (% pred)	66.3 ± 16.8	63.4 (54.6, 81.3)	--	--	--	--

Criteria or calculation of predicted values by [28]. Comparison to predicted values performed by one-sample t-test with mean difference and Hedges g, or ^a^Wilcoxon Signed rank test with median difference and correlation coefficient r reported. ^b^peak HR was not captured in one participant (*n* = 15). --, not known or not performed

#### Relationship between CPET outcomes and 6MWT distance

**Fig. 2:**
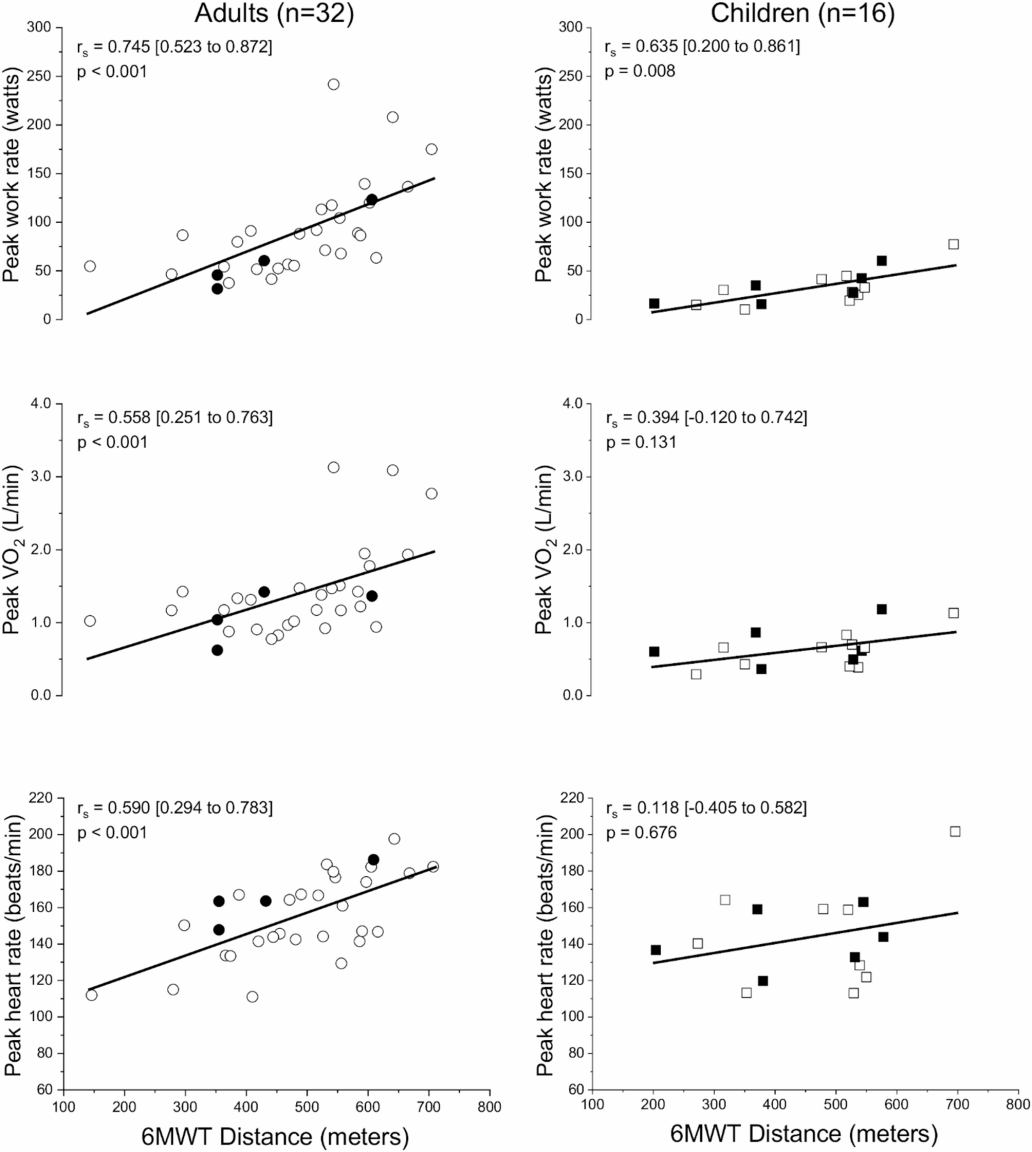
Spearman’s correlation with 95% CI [lower to upper] bounds between indices at peak exercise and 6MWT distance at baseline (month-0) for adults (circle symbols; left panels) and children (square symbols; right panels). Symbols in black denote participants with recessive mode of inheritance

Performance on the CPET vs. total distance walked on the 6MWT are shown in Fig. 2. Among the adult population, a positive and moderately strong correlation between indices of cardiorespiratory performance and the 6MWT distance (*p* < 0.001, 0.558 ≤ r_s_ ≤ 0.745) was observed. In children, however, only work rate had a moderately strong correlation with distance walked (*p* = 0.008, r_s_ = 0.635), and fair to poor correlation with peak VO_2_ (*p* = 0.131, r_s_ = 0.394) and HR (*p* = 0.676, r_s_ = 0.118), respectively.

The relationship between total distance walked on the 6MWT and indices at AT for work rate, VO_2_ and heart rate in adults are shown in Supplementary Fig. 3. The correlation between work rate at the AT and total distance walked was moderately strong (*p* < 0.001, r_s_ = 0.736), while only fair for VO_2_ (*p* = 0.021, r_s_ = 0.419) and HR (*p* = 0.080, r_s_ = 0.326) at the AT with the 6MWT distance.

For adults, a multiple linear regression model that included 6MWT distance, BMI and sex (Supplementary Table 2), accounted for 64% of the variance in predicting absolute peak VO_2_ (adjusted R^2^ = 0.635, F(3,28),  = 18.979, *p* < 0.001). The standard error of the estimate (SEE) was 0.370 L/min, representing 26.5% of the mean peak VO_2_. Age did not improve the model, therefore was not included. Among children, a multiple linear regression model including age, 6MWT distance and sex (Supplementary Table 3), accounted for 76% of the variance in predicting absolute peak VO_2_ (adjusted R^2^ = 0.764, F(3,12),  = 17.209, *p* < 0.001) and the SEE was 0.126 L/min or 20.9% of the mean peak VO_2_. BMI did not improve the model, therefore was not included.

### CPET performance changes over time: baseline to month six

Mean difference (month-6 minus month-0) in CPET parameters for adults and children are presented in Table 5, with AT changes in adults shown in Supplementary Table 4. The CPET was completed by 19 adults at the 6-month time point (41% drop-out due to early study closure and withdrawals) [16], while only one child did not complete the CPET at the follow-up time point (6% drop-out). Over the course of 6 months, cardiorespiratory performance in both adults and children remained stable, with small effect sizes (0.161 ≥ *p* ≥ 0.894; -0.321 ≤ g ≤ 0.213, *r* = 0.042). As reported previously [25], the 6MWT distance was also observed to be unchanged at month six from baseline (mean difference [95% CI], *p*-value, Hedge’s g; Adults: +7.5 m [-8.2 to 23.1], *p* = 0.332, g = 0.214; Children: -3.3 m [-40.4 to 33.7], *p* = 0.850, g = -0.047). Correlations between CPET variables and 6MWT distance at month six varied from poor to very strong, similar to baseline for adults (0.001 ≤ *p* ≤ 0.019, 0.533 ≤ r_s_ ≤ 0.886) and children (0.001 ≤ *p* ≤ 0.524, 0.179 ≤ r_s_ ≤ 0.843). (Supplementary Fig. 4).

**Table 5:** Peak CPET changes over the course of 6 months in adults and children

	Adults (*n* = 19)		**Effect Size (g or r**^**a**^)		Children (*n* = 15)		**Effect size**,** g**
	**Mean/Median**^**a**^ **Diff [95% CI]**	**p-value**			**Mean Diff [95% CI]**	**p-value**	
Time to peak, sec	-3 [-34 to 27]^a^	0.856	0.042^a^		-4 [-58 to 51]	0.894	-0.033
Peak RER	-0.01 [-0.07 to 0.05]	0.805	-0.055		0.03 [-0.05 to 0.11]	0.426	0.200
Peak WR, watts	0.4 [-4.8 to 5.6]	0.880	0.034		1.4 [-3.9 to 6.8]	0.577	0.140
Peak VO_2_, L/min	-0.07 [-0.18 to 0.03]	0.161	-0.321		-0.02 [-0.10 to 0.07]	0.662	-0.109
Peak HR, beats/min	1 [-6 to 8]	0.834	0.047		-1 [-13 to 11]^b^	0.841	-0.052
Peak VE, L/min	3.2 [-3.0 to 9.5]	0.388	-0.194		0.2 [-3.2 to 3.7]	0.890	0.034
Peak RR, breath/min	1.9 [-2.2 to 6.0]	0.243	-0.265		4.3 [-6.2 to 14.8]	0.397	0.213

Comparison performed by paired-samples t-test with mean difference and Hedges’s g or ^a^related samples Wilcoxon signed rank test with median difference and correlation coefficient r reported. ^b^peak HR was not captured in one child at baseline (*n* = 14)

## Discussion

Individuals with *RYR1*-RM are reported to exhibit exercise intolerance, which can be partly attributed to disease pathophysiology resulting in skeletal muscle weakness and hypotonia, and adaptation to a sedentary lifestyle [32]. In this study, adults and children with *RYR1*-RM performed a standardized cardiopulmonary exercise test to describe the exercise capacity and response in this population, which were compared to expected normal values. Evaluation of change in exercise capacity over six months was also reported. Outcomes from the CPET were also examined in relation to the 6MWT, a field test widely used in populations with neuromuscular disorders [33].

The integration of the cardiovascular, pulmonary, and muscular systems during a CPET allows a clinical evaluation of abnormal exercise capacity [20]. Among adults with neuromuscular disorders, specific criteria can be used to distinguish those that gave maximal effort on the CPET [22]. In this study, all participants that performed the CPET were included as exercise capacity and cardiorespiratory related-responses have not been previously described among a *RYR1*-RM cohort. Most adults with *RYR1*-RM reached ≥1.10 RER (23 of 32 adults), a target used among healthy individuals [26] and clinical patients [34] to demonstrate sufficient effort on the CPET. Of these 23 participants, only 6 adults reached a peak VO_2_ ≥85% of predicted. Thereby, a total of 81% of adults showed low exercise tolerance. Further, more than a third of all participants had severely limited exercise capacity of < 50% predicted peak VO_2_, which was unrelated to test effort since 8 of the 12 participants in this category had RER > 1.10. The dynamic profiles of the metabolic, cardiovascular and ventilatory systems during the CPET revealed a normal metabolic response with appropriate ventilatory function, yet an exaggerated cardiovascular response for the given metabolic demand in certain individuals. Particularly in adults with severely reduced exercise capacity, a greater than expected reliance on the cardiovascular system was observed, especially as compared to those with preserved exercise capacity (Supplemental Fig. 1). A low peak VO_2_ in the presence of a normal peak HR may result in “hyperdynamic circulation” and has been previously observed in patients with myopathic disorders [19]. The exaggerated slope of ∆HR/∆VO_2_ may reflect impairments to peripheral (i.e., muscle oxygen extraction) and/or central (i.e., stroke volume) O_2_ delivery [24], and further investigations are needed in the *RYR1*-RM population.

**Fig. 3:**
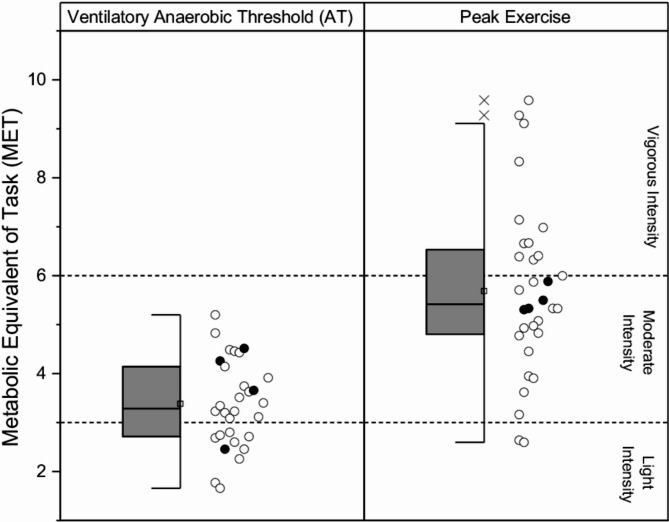
The Metabolic Equivalent of Task (MET) at the ventilatory anaerobic threshold (AT) and peak exercise for adults with *RYR1*-RM. Standard box plot with ranges (Q1 to Q3), median (line), mean (square symbol), 1.5 times interquartile range (whiskers) and outliers (diagonal cross symbols) shown, with individual data points (circle symbols). Dashed lines demarcate level of absolute intensity in MET for light (< 3 MET), moderate (≥ 3 and < 6 MET), and vigorous (≥ 6 MET) per [48]. Symbols in black denote adult participants with recessive mode of inheritance

A common complaint and feature of *RYR1*-RM is severe fatigue [35]. During a CPET, the AT identifies the exercise intensity when anaerobic metabolism is increasingly relied on to meet higher energy needs [20]. As anaerobic metabolism is considered an inefficient means of energy production [19] with generation of by-products associated with muscle fatigue [36], the AT can serve as a marker of an individual’s susceptibility to fatigue. In other myopathies such as McArdle’s disease however, the AT may not be discernable [19]. The AT was identified in 30 adults, of which 73% were observed to be below normal. This suggests that *RYR1*-RM adults have a lower threshold to perform activities prior to the onset of physical fatigue. The metabolic equivalent of task (MET) at the AT was 3.4 MET (Fig. 3), representing activities at the lower end of moderate intensity (e.g. grocery shopping has a 3.3 MET value [37]), which may be fatiguing for some of these individuals to perform. Coupled with an average 5.7 MET at peak capacity, most of these adults with *RYR1*-RM are likely to exert maximal effort to perform activities typically considered as moderate intensity (e.g. carrying groceries upstairs at 5.3 MET [37]).

Exercise performance on the CPET in children with *RYR1*-RM was also lower when compared to normative ranges in children of similar age, sex, BMI and race [28]. Of note, only a small number of children (~ 30%) managed to sustain exercise for at least 8 min and/or achieve an RER equal to or greater than 1.10. Low RER values can occur among younger children at peak exercise [38], with higher RER values being observed with increasing age [28]. In this study, short exercise duration or low RER were not restricted to the younger participants, suggesting that perhaps the ramp rate of 5 watts/min on a pediatric cycle ergometer was still too aggressive for children with *RYR1*-RM. While it is difficult to discern whether early test termination on the CPET may accurately reflect the cardiorespiratory capacity of children with *RYR1-*RM, the reproducibility of these findings at the 6-month timepoint supports the interpretation that participants likely cycled to the limit of their tolerance.

Cardiorespiratory fitness outcomes were also found to be directly related to total 6MWT distance, in both adult and pediatric populations. For adults, this relationship was observed at both peak exercise and AT for work rate and VO_2_. In children, only peak work rate achieved on the CPET showed a moderately strong direct relationship with distance walked on the 6MWT. To examine whether the 6MWT distance together with other patient demographics could predict peak VO_2_, separate models for adults and children with an explained variance of 64% and 76%, respectively, was observed (Supplementary Tables 2 and 3). However, the SEE was considered high (27% and 21% of the respective mean peak VO_2_ in adults and children), and while consistent with reported SEE values among pulmonary and cardiac patients [39, 40], these suggest limited application at the individual level. All participants that performed the CPET were also included in the models as specific criteria to confirm “maximal” effort has not been established in this population, which further reduces the accuracy to predict peak VO_2_. Therefore, the 6MWT distance may not be able to predict an individual participant’s peak VO_2_ accurately, yet may still provide generalized information on functional performance and overall group exercise capacity. A consideration is in children, where the 6MWT was an easier test to follow and conduct, suggesting that the 6MWT may still be an appropriate test among children with *RYR1*-RM in evaluating physical capacities. Further studies would be needed in larger numbers to confirm these results in both adults and children with *RYR1*-RM.

*RYR1*-RM are often slowly progressive and previously reported to be functionally stable over six months [16]. Consistent with previous observations [16, 41], overall cardiorespiratory performance did not change over the six-month period in either adults or children (including AT in adults). Of note, the ability to ambulate independently was a criterion to participate in the parent study. Although severe dominant cases of *RYR1*-RM have been reported, recessive cases are typically of greater severity, with more widespread fibro-fatty muscle replacement [12, 42]. In the present study, this was not observed as recessive cases did not primarily display the lowest exercise capacities, suggesting that high disease activity may have a role in reduced exercise tolerance among *RYR1*-RM, however the ambulatory inclusion criterion must also be considered.

Exercise is a particular concern for *RYR1*-RM patients and their care providers [43] since exercise could trigger rhabdomyolysis [44], orthopedic issues may affect mobility, and weakened muscles may impair respiratory function [1]. However, expert consensus for CMyo [1] and muscular disorders [45] recommend regular exercise, which highlights the importance of encouraging exercise adoption among the *RYR1*-RM population. Physical exercise can improve function, support independence, and minimize the deleterious effects of low physical activity [1]. While optimized exercise prescriptions for *RYR1*-RM are currently unknown, exercise should be individualized and adapted to progressively increase the components known to produce a training effect (i.e., frequency, intensity, time, and type) [45], with the added caution to limit exercise to an intensity that does not result in muscular fatigue or soreness [1]. High intensity exercise has been associated with RyR1 fragmentation and lower muscular force production in untrained healthy adults [46], while fatigue was cited as the main cause of drop-outs from an exercise training program of moderate-intensity in adults with CMyo [11]. Additionally, exertional rhabdomyolysis is a well-established concern in this patient population, especially in the context of prolonged exercise in an environment with elevated temperature [14, 44]. While the adults and children with *RYR1*-RM in this study were able to perform and tolerate acute bouts of short and relatively high exercise intensity without any related adverse events, careful supervision of any sustained or vigorous exercise is needed owing to possible susceptibility of this patient population to exercise-induced rhabdomyolysis and malignant hyperthermia [14].

Performance on a CPET in adults revealed some avenues that may deepen our understanding of reduced exercise capacity among *RYR1*-RM, which can be leveraged in future studies for targeted rehabilitation. Exercise prescriptions have been recommended to be individualized for muscular disorders [45], and findings presented in this study may provide further guidance for clinicians and physical therapists to support their patients with *RYR1*-RM towards long-term improvements in functional capacity. There is a clear need for further studies to inform the effective dosage and type of exercise [45] that balances the benefits and risks in persons with *RYR1*-RM.

### Limitations

Our analyses would benefit from additional samples of recessive cases as the low number limited the ability to form any conclusions related to recessive *RYR1*-RM. As *RYR1*-RM is a congenital myopathy that commonly presents at birth or early childhood, the limited CPET data for a relatively small cohort of children restricts the ability to generalize these findings to the pediatric *RYR1*-RM population. A longer natural history follow-up with a larger sample size to include more severely affected individuals is needed for clinical trial readiness in this slowly progressive, debilitating, and heterogeneous group of disorders. The CPET is not reflective of a participants’ free-living physical activity, therefore collection of daily activity information with a wearable accelerometry device may provide a more clinically meaningful outcome to inform future clinical trial design for investigational products targeting physical fatigue. Such efforts are ongoing in the U.S. (e.g. NCT06287762) and Europe (e.g. NL-OMON56064). It is also possible that local differences in muscle metabolism and degree of muscle replacement with non-contractile tissue could be a contributing factor to inter-individual variability over the six-month period. However, such possibility is minimal insofar as this was primarily a lower extremity exercise test involving large skeletal muscle groups. Finally, three individuals with *RYR1*-related exertional rhabdomyolysis were included in the dominant cases. The phenotypic trajectory of *RYR1*-related exertional rhabdomyolysis includes progressive muscle weakness with age [47], and while this group is affected to a lesser extent vs. CMyo phenotypes, they still report greater fatigue and functional difficulties compared to otherwise healthy individuals [35].

Strengths of this study include an objective approach for the longitudinal measurement of peak exercise capacity and AT in this rare neuromuscular disease cohort. Specific outcomes of peak exercise capacity were positively related to the field measure of 6MWT in both adults and children, however, predictive models are currently insufficient for accurate peak VO_2_ values among individual cases.

## Conclusions and clinical implications

Exercise capacity is diminished in adults and children with *RYR1*-RM, however, remains stable over six months. Lower exercise capacity and AT translates to physical fatigue being experienced by persons with *RYR1*-RM undertaking common everyday tasks. Indications of an exaggerated cardiac response despite low peak VO_2_ in certain *RYR1*-RM adults are consistent with observations in other muscular disorders [19]. The 6MWT distance showed the highest association with peak work rate on the CPET in both adults and children with *RYR1*-RM. While mode of inheritance in *RYR1*-RM may have an impact on the observed findings, the small number of recessive cases in this cohort limits the ability to explore this further. Exercise capacity testing could reveal avenues for in-depth studies of muscular dysfunction [19] affecting individuals with *RYR1*-RM and could inform the development of tailored exercise regimens.

## Supplementary Information

Below is the link to the electronic supplementary material.

**Supplementary Material 1: Additional File 1**. Supplementary Table (1) Supplementary Fig. 1 Supplementary Fig. 2. Supplementary Fig. 3. Supplementary Table (2) Supplementary Table (3) Supplementary Table (4) Supplementary Fig. 4

## Data Availability

The deidentified data related to the results reported in this article are available from the corresponding author on reasonable request.
