# Supplementary material for: Exercise capacity in RYR1-related myopathies
Source: Orphanet J Rare Dis. 2025 Sep 24;20:485. doi: 10.1186/s13023-025-04013-7 (PMC12461967; doi:10.1186/s13023-025-04013-7)
Supplement: Supplementary file 1 — Supplementary Material 1: Additional File 1. Supplementary Table (1) Supplementary Fig. 1 Supplementary Fig. 2. Supplementary Fig. 3. Supplementary Table (2) Supplementary Table (3) Supplementary Table (4) Supplementary Fig. 4 [file 13023_2025_4013_MOESM1_ESM.docx]

**Supplementary Material**

**Supplementary Table 1** Slopes for Metabolic, Cardiovascular and Ventilatory Indices (n = 32)

|  | **Mean ± SD** | **Median (Q1, Q3)** | **Expected value** | **Mean/Median^a^ Diff [95% CI]** | **p-value** | **Effect size (g or r^a^)** |
| --- | --- | --- | --- | --- | --- | --- |
| **Metabolic Indices** | | | | | |  |
| ∆VO_2_/∆WR (ml/min/watt) | 11.1 ± 1.7 | 11.1 (9.5, 11.9) | 10.6 ± 0.6 [22] | 0.4 [0.0 to 1.0]^a^ | 0.054 | 0.340^a^ |
| ∆VO_2_/∆WR (% pred) | 105 ± 15 | 105 (95, 115) | -- | -- | -- | -- |
| **Cardiovascular Indices** | | | | | |  |
| ∆HR/∆VO_2_ (beat/min⋅L/min) | 71 ± 24 | 69 (53, 84) | 64 ± 17 [22] | 7 [-1 to 14] | 0.079 | 0.314 |
| ∆HR/∆VO_2_ (% pred) | 114 ± 34 | 106 (90, 129) | -- | -- | -- | -- |
| **Ventilatory Indices** | | | | | |  |
| ∆Ve/∆VCO_2_ (L/min⋅L/min) | 25 ± 3 | 26 (23, 28) | 27 ± 2 [22] | -1 [-3 to 0]^a^ | 0.027 | 0.390^a^ |
| ∆Ve/∆VCO_2_ (% pred) | 94 ± 12 | 98 (86, 103) | -- | -- | -- | -- |
| --, not known or not performed. Calculation of predicted values by [22]. Comparison to predicted values performed by one-sample t-test with mean difference and Hedges’s g, or ^a^Wilcoxon signed rank test with median difference and correlation coefficient r | | | | | | |

**

**

**Supplementary Fig. 1** Spearman’s correlation with 95% CI [lower to upper] bounds between peak VO_2_ and slope of HR/VO_2_ in percent predicted. Symbols in black denote adult participants with recessive mode of inheritance. Level of severity based on %VO_2_ predicted per Glaab et al. [23]


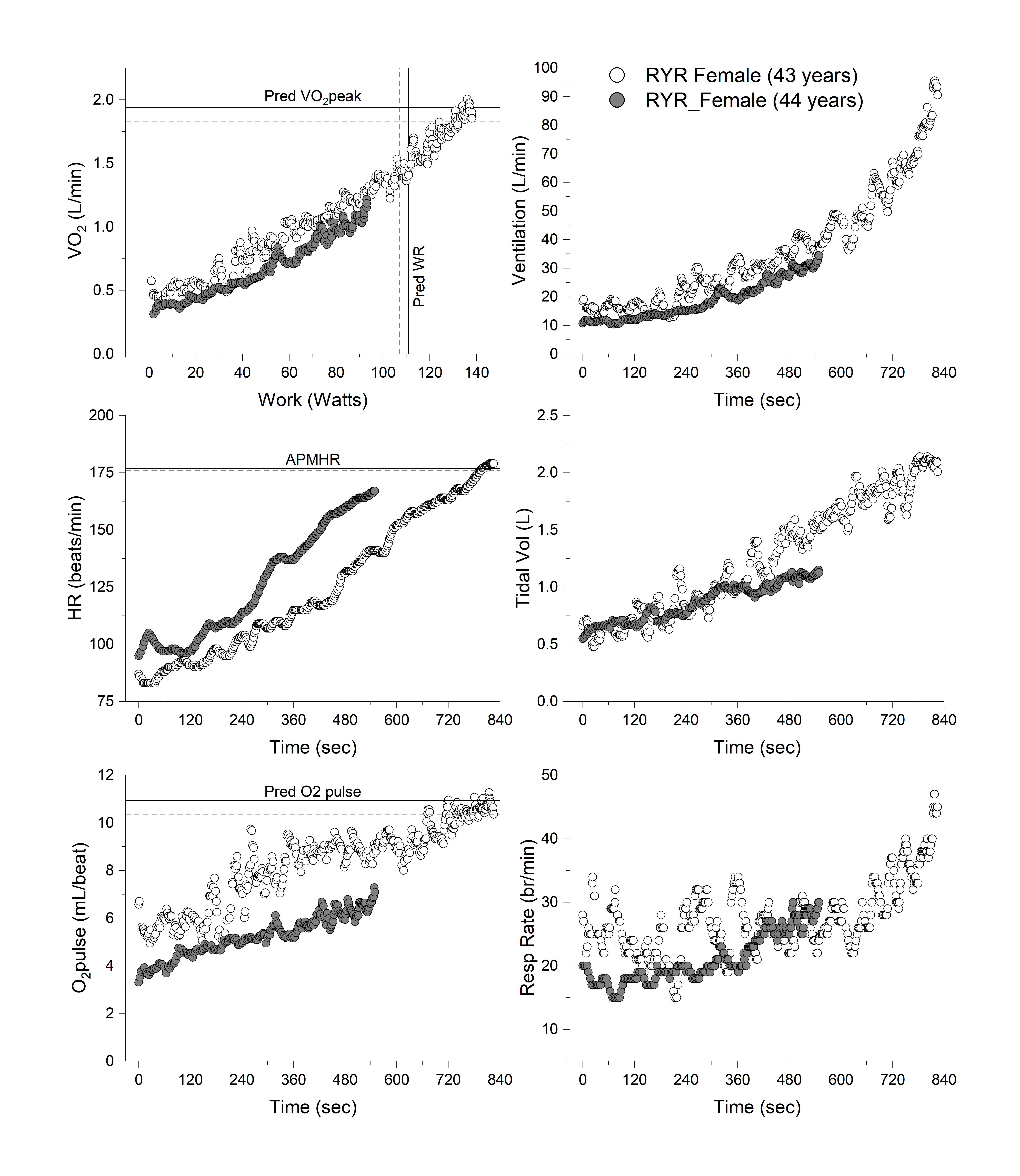


**Supplementary Fig. 2** Raw responses to the CPET for two females of similar age with *RYR1*-RM. Female that responded as expected (white symbols) and reached her expected values (solid line) vs. female that did not reach her expected values (grey symbols, dashed line)


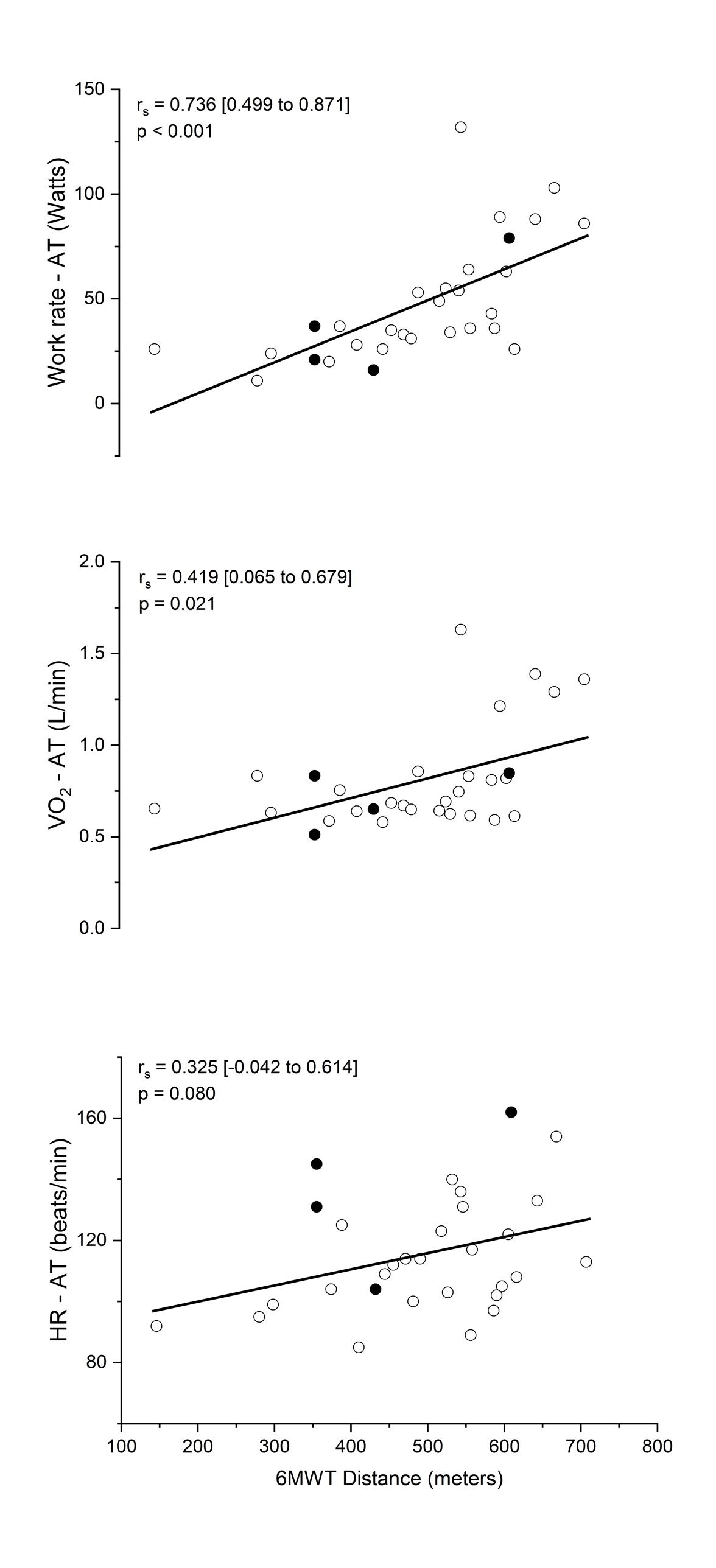


**Supplementary Fig. 3** Spearman’s correlation with 95% CI [lower to upper] bounds between indices at the AT and 6MWT distance at baseline (month-0) for adults. Symbols in black denote adult participants with recessive mode of inheritance

**Supplementary Table 2** Multiple linear regression model in adults for predicting peak VO_2_ in L/min

| **Variable** | **Unstandardized coefficient B** | **95% Confidence Interval** | **p-value** |
| --- | --- | --- | --- |
| Constant | -1.125 | -1.937 to -0.313 | 0.008 |
| 6MWT distance | 0.003 | 0.002 to 0.004 | < 0.001 |
| BMI | 0.047 | 0.028 to 0.066 | <0.001 |
| Sex | -0.425 | -0.697 to -0.153 | 0.003 |

6MWT distance in meters, BMI in kg/m^2^; coding for sex: 0 = male; 1 = female

**Supplementary Table 3** Multiple linear regression model in children for predicting peak VO_2_ in L/min

| **Variable** | **Unstandardized coefficient B** | **95% Confidence Interval** | **p-value** |
| --- | --- | --- | --- |
| Constant | -0.362 | -0.717 to -0.007 | 0.046 |
| Age | 0.080 | 0.050 to 0.110 | < 0.001 |
| 6MWT distance | 0.001 | 0.00 to 0.001 | 0.012 |
| Sex | -0.186 | -0.329 to -0.044 | 0.015 |

6MWT distance in meters, Age in years; coding for sex: 0 = male; 1 = female

**Supplementary Table 4** Ventilatory Anaerobic Threshold (AT) changes over the course of 6 months in adults (n=19)

|  | **Mean/Median^a^ Difference [95% CI]** | **p-value** | **Effect size (g or r^a^)** |
| --- | --- | --- | --- |
| WR (watts) | 2.4 [-3.4 to 8.2] | 0.404 | 0.188 |
| VO_2_ (L/min) | -0.01 [-0.08 to 0.07] | 0.862 | -0.039 |
| HR (beats/min) | 1.4 [-3.8 to 6.6] | 0.571 | 0.127 |
| VE/VCO_2_ | 0.0 [-0.5 to 1.0]^a^ | 0.321 | 0.228^a^ |

Comparison performed by paired-samples t-test with mean difference and Hedges’s g or ^a^related samples Wilcoxon Signed rank test with median difference and correlation coefficient r reported

**

**

**Supplementary Fig. 4** Spearman’s correlation with 95% CI [lower to upper] bounds between indices at peak exercise and 6MWT distance at follow-up (month-6) for adults (circle symbols; left panels) and children (square symbols; right panels). Symbols in black denote participants with recessive mode of inheritance
